# Outcomes of 25-gauge pars plana vitrectomy alone with air tamponade for the management of rhegmatogenous retinal detachment with inferior breaks

**DOI:** 10.1186/s12886-022-02445-4

**Published:** 2022-05-12

**Authors:** Yongping Tang, Bo Lin, Jing Chen, Daosen Chen, Ronghan Wu

**Affiliations:** 1 Eye Hospital and School of Ophthalmology and Optometry, Wenzhou Medical University, Wenzhou, Zhejiang China; 2 National Clinical Research Center for Ocular Diseases, Wenzhou, Zhejiang China; 3 Yuying Children’s Hospital, The Second Affiliated Hospital of Wenzhou Medical University, Wenzhou, Zhejiang China

**Keywords:** Rhegmatogenous retinal detachment, Inferior retinal break, Air tamponade, Pars plana vitrectomy

## Abstract

**Background:**

This study was investigated the surgical outcomes of primary rhegmatogenous retinal detachment (RRD) with inferior retinal breaks (IRBs) that were repaired by 25-gauge pars plana vitrectomy (PPV) with air tamponade.

**Methods:**

This retrospective review included 81 consecutive patients who had RRD with IRBs and underwent PPV with air tamponade in our hospital from January 2017 to January 2020. The main outcomes were single surgery anatomical success (SSAS) rate, postoperative best-corrected visual acuity (BCVA), and complications.

**Results:**

The patient population consisted of 29 women and 52 men (mean age, 52.12 years); the mean follow-up interval was 8.88 months. The mean number of affected quadrants was 1.65 (range, 1–4 quadrants) and the mean number of breaks was 3.25. A single break was present in 20 cases (24.7%); two to 10 breaks were present in 61 (75.3%) cases. The SSAS rate was 91.36% (74/81) and the final anatomical success rate was 96.30% (78/81). More than half of the patients had BCVA < 0.3 logarithm of the minimum angle of resolution at the last follow-up. Axial length and patient age were candidate risk factors for redetachment (axial length, *p* = 0.03; age, *p* = 0.002). Postoperative complications included macular epiretinal membrane formation in one patient, lens opacity in three patients, and clinically significant macular edema in one patient.

**Conclusions:**

PPV with air tamponade may be effective for the treatment of primary RRD with IRBs. Extensive preoperative discussion may be necessary for young patients and patients with particularly long axial length.

## Background

Retinal detachment is an important cause of vision loss. Because of improvements in surgical techniques, the rate of successful anatomical retinal reattachment in primary retinal detachment is high (> 80%) [1]. However, the management of rhegmatogenous retinal detachment (RRD) with inferior retinal breaks (IRBs) via pars plana vitrectomy (PPV) surgery remains challenging because of insufficient intraocular tamponade (e.g., long-acting gases, silicone oil [SO], and air). Thus, other strategies have been proposed, including the combined use of scleral buckling (SB) and PPV, as well as the use of heavy silicone oil or perfluoro-n-octane as a tamponade agent [2]. Those strategies can cause substantial complications: combined SB may lead to choroidal hemorrhage, myopia, diplopia or ptosis [3]. Long-acting gases can disturb the vitreous, thus increasing the risks of elevated intraocular pressure (IOP), lens opacity, proliferative vitreoretinopathy (PVR) and new or missed breaks [4]. SO, heavy SO and perfluoro-n-octane may cause high intraocular pressure (IOP), lens opacity, corneal degeneration and retinal toxicity. Furthermore, secondary surgery is needed to remove SO, heavy SO and perfluoro-n-octane; such surgery increases the financial burden carried by patients [5].

Although air tamponade is rapidly absorbed and may not provide a substantial tamponade effect, previous studies have demonstrated that this approach can be used to successfully repair RRD with superior retinal breaks [6, 7]. There remains controversy concerning the advantages of air tamponade for treatment of RRD with IRBs, compared with long-acting gases [8–11]. In China, long-acting gases are not commercially available because of legislative changes made in 2016; thus, surgeons can use either air or SO as the tamponade for RRD during PPV, considering the findings in the preoperative evaluation and clinical examination [10]. Importantly, many patients with noncomplicated RRD including IRBs have undergone PPV plus air tamponade since 2016 in China.

Here, we reviewed clinical data from patients with primary RRD and causative retinal breaks located between 5- and 7-o'clock, all of whom underwent PPV with air tamponade. We evaluated the anatomical and functional outcomes of vitrectomy with air tamponade in the treatment of primary RRD with IRBs, with the expectation that an IRB was a risk factor for redetachment.

## Methods

In this retrospective analysis, we collected clinical data from patients who had undergone 25-gauge PPV with air tamponade for primary RRD in our hospital, during the period from January 2017 to January 2020. Exclusion criteria were giant retinal tears, retinal dialysis, tractional, serous retinal detachment, posttraumatic retinal detachment, PVR of grade ≥ C2 and history of PPV.

Of the 245 patients who underwent PPV with air tamponade during the inclusion period, 81 patients had retinal breaks located between 5 and 7 o’clock. Each patient received information about the procedure, then provided written informed consent to undergo surgery. This study was conducted in accordance with the Declaration of Helsinki and approved by the Ethics Committee of the Eye Hospital, School of Ophthalmology and Optometry, Wenzhou Medical University (2020–086-K-78).

Pre-, intra- and postoperative findings were documented. Preoperative evaluations included best-corrected visual acuity (BCVA) assessment, IOP assessment, slit-lamp biomicroscopy, fundus examination with an indirect ophthalmoscope, optical coherence tomography and ocular B-scan ultrasonography. Surgical records were consulted to determine the characteristics of detached retina and retinal breaks, including the number of affected quadrants, macular status, the number and location of breaks and retinal degeneration. BCVA assessment, IOP assessment, slit-lamp examination and fundus examination were performed at each postoperative visit. Anatomical success was defined as complete retinal attachment at the last follow-up (without SO retained). BCVA was measured using a Snellen eye chart, and then converted to the logarithm of the minimum angle of resolution (logMAR). BCVA values of counting fingers, hand movement, light perception and no light perception were converted to logMAR values of 1.85, 2.3, 2.6 and 2.9, respectively [12]. Additionally, breaks were classified as small (< 0.5 disc diameter [DD]), medium (0.5–2.0 DD), or large (> 2 DD) according to their longest meridian in relation to the optic disc diameter [13].

All procedures were performed under retrobulbar anesthesia by a single surgeon (R.W.). The Constellation Vision System (Alcon Laboratories, Inc., Fort Worth, TX, USA) was used to perform 25-gauge three-port PPV with a wide-angle non-contact viewing system (Resight®; Carl Zeiss Meditec AG, Jena, Germany). Phacoemulsification and intraocular lens implantation were performed for patients with obvious lens opacity and patients aged > 50 years. Intraoperative scleral indentation was performed to trim the vitreous base, identify retinal breaks and remove traction from retinal tears. Complete air-fluid exchange was conducted; subretinal fluid was aspirated through the breaks with a flute needle to ensure reattachment. Retinopexy with laser photocoagulation and/or cryotherapy were performed. IOP was assessed by tactile examination. When necessary, additional air was injected through the pars plana to adjust the IOP. All patients were instructed to assume a prone position on the first day after surgery; they were then instructed to assume a prone or lateral recumbent posture (i.e., “support-the-break positioning”) for 7 days. Tobramycin and dexamethasone eye drops were administered for 1 week postoperatively; 1% fluorometholone eye drops and levofloxacin eye drops were administered thereafter until 4 weeks postoperatively.

LogMAR BCVA values were used for analysis. IOP and BCVA values were compared between preoperative and follow-up time points by using paired-samples t-tests. Multivariate analyses were conducted to analyze independent risk factors for redetachment. *p-*values < 0.05 were considered statistically significant. All statistical analyses were performed using SPSS version 16.0 (IBM Corp., Armonk, NY, USA).

## Results

During the inclusion period, 245 patients with primary RRDs were treated with PPV with air tamponade in our hospital. Of those patients, 81 with IRBs (between 5 and 7 o’clock) were enrolled in this study. The demographics and preoperative clinical characteristics of the enrolled patients are shown in Table 1. Posterior vitreous detachment was diagnosed using ocular B-scan ultrasonography before surgery; the diagnosis was confirmed during surgery. In this study, 32 eyes had IRBs alone, while 49 eyes had both superior retinal breaks and IRBs. The characteristics of retinal breaks and holes are summarized in Table 2.

**Table 1:** Demographic and clinical characteristics of patients suffered rhegmatogenous retinal detachment with inferior retinal breaks

Factors	
Male (Female)	52(29)
Mean age (Mean ± SD, years)	52.12 ± 11.75
Duration of symptoms (Mean ± SD, days)	16.48 ± 18.03
Eye (OD/OS)	41/40
Mean axial length (Mean ± SD, mm)	25.52 ± 2.34
Number of axial lengths over 26 mm	30 (37.03%)
Number of macula-off detachments	66 (81.48%)
Mean number of quadrants affected (Mean ± SD)	1.65 ± 0.73
Presence of preoperative lattice degeneration	53 (65.43%)
Phakic eyes	75 (92.59%)
Number of PEA and IOL implantation	46 (56.79%)
Retinopexy	
Laser	70 (86.42%)
Cryotherapy	1 (1.23%)
Laser and cryotherapy	10 (12.35%)

*PEA* Phacoemulsification aspiration, *IOL* Intraocular lens, *SD* Standard deviation

**Table 2:** Characteristics of retinal breaks (intraoperative observation)

Characteristics	
Mean number of retinal breaks (Mean ± SD)	3.25 ± 2.65
Retinal breaks, size	
Small breaks	86(32.7%)
Medium breaks	110(41.8%)
Large breaks	67(25.5%)
Single/ multiple breaks (cases)	20/61
Retinal breaks, position	
5–7 o’clock	144(54.8%)
Number of horseshoe breaks/ atrophic holes	167/96

**Fig. 1:**
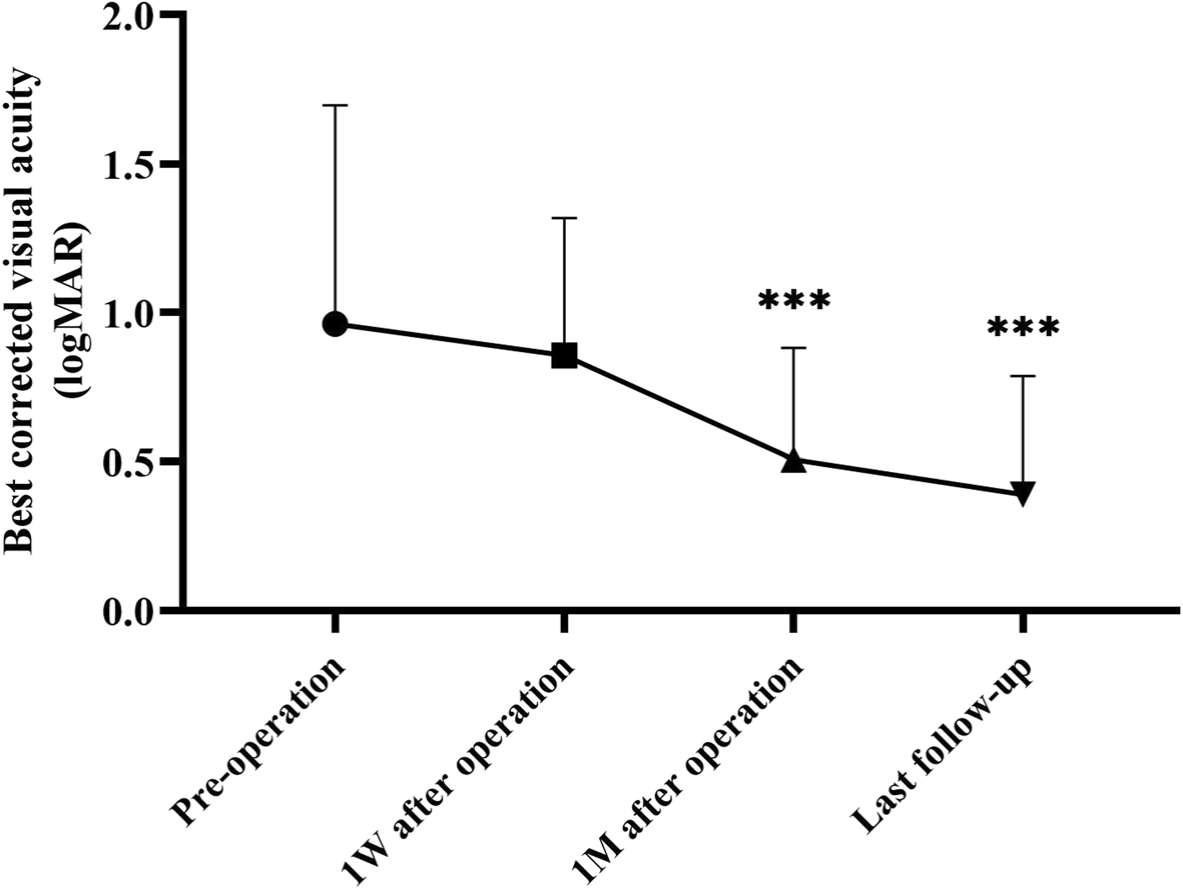
Visual outcomes of patients who underwent PPV plus air tamponade for RRD. Postoperative BCVA improved from 1 week after surgery onwards; significant differences were found at 1 month postoperatively and at the last follow-up, compared with preoperative BCVA (***: *P* < 0.001, W = week, M = month). (RRD, rhegmatogenous retinal detachment; PPV, pars plana vitrectomy; BCVA, best-corrected visual acuity)

The mean follow-up interval was 8.88 months. The SSAS rate was 91.36% (74/81) and the final anatomical success rate was 96.30% (78/81). All eyes demonstrated BCVA improvement after surgery (Fig. 1). Postoperative BCVA improved from 1 week after surgery onwards; significant differences were found at 1 month postoperatively and at the last follow-up, compared with preoperative BCVA (*p* < 0.001). Overall, 46 (56.79%) patients had BCVA < 0.3 logMAR at the last follow-up. The IOP value at each postoperative time point was higher than the preoperative value (*p* < 0.001). Nine patients had IOP > 21 mmHg (range, 22.2-45.2 mmHg) at 1 week postoperatively; all patients underwent application of antihypertensive topical medication, and their IOP values returned to normal at 1 month postoperatively.

After surgery, epiretinal membrane developed in one eye; membrane peeling surgery was then performed. Three patients developed lens opacity at 4 to 8 months after surgery; phacoemulsification and intraocular lens implantation were then performed. One patient developed substantial macular edema, which was treated with intravitreal triamcinolone acetonide. Two patients underwent outpatient laser photocoagulation at 1 week postoperatively; this enhanced chorioretinal adhesion around the breaks.

The details of patients who experienced redetachment after primary PPV are shown in Table 3. Retinal redetachment occurred in seven eyes; all patients with redetachment had undergone PPV with SO tamponade. We analyzed the possible risk factors for redetachment (Table 4). The variables tested included the following: age, axial length, number of retinal breaks, number of quadrants affected, macula-off detachment and number of preoperative lattice degenerations. Univariate analysis result showed that axial length and patient age were candidate risk factors for redetachment (axial length, *p* = 0.03; age, *p* = 0.002, Table 4). Thus, we further did multivariate logistic regression analysis with the two candidate risk factors (axial length and age) showed that age of patient was an independent risk factor for redetachment in our group of study population (*p* = 0.04, odds ratio = 0.89).

**Table 3:** Clinic characteristics of patients with redetachment after primary PPV

Factors	Case1	Case2	Case3	Case4	Case5	Case 6	Case 7
Age (years)	28	40	38	44	32	65	24
Gender	M	F	M	F	M	M	M
Duration of symptoms (days)	3	20	4	10	6	60	10
Axial length (mm)	30.21	25.53	29.9	24.5	26.03	25.17	29.81
Lens status	Phakia	Phakia	Phakia	Phakia	Phakia	Phakia	Phakia
PEA and IOL implantation	NO	NO	NO	NO	NO	YES	NO
Cause of redetachment	New breaks	Unsealed breaks	PVR	reopening of break	Unsealed breaks	new breaks	new breaks
Duration until redetachment (weeks)	1	1	24	4	1	2	1
Intraocular tamponade	SO	SO	SO	SO	SO	SO	SO
Duration of SO tamponade(months)	4	5	Retained	Retained	Retained	4	4

*M* Male, *F* Female, *PVR* Proliferative vitreoretinopathy, *PEA* Phacoemulsification aspiration, *IOL* intraocular lens, *SO* Silicone oil

**Table 4:** Analysis of risk factors for redetachment

	Success (*N* = 74)	Failure (*N* = 7)	*p*-value
Age (years)	52.34	38.29	**0.002**
Axial length (mm)	25.36	27.31	**0.03**
Macula-off detachment	59 (78%)	7(100%)	0.34
Number of retinal breaks	3.20	3.71	0.63
Number of quadrants affected	2.39	2.43	0.92
Number of preoperative lattice degenerations	1.05	1.29	0.56

## Discussion

RRD with IRBs remains difficult to manage solely with PPV with intraocular tamponade, particularly when the tamponade comprises gas or air [14–16]. With the increasing use of endolaser treatment and drainage of subretinal fluid during PPV, chorioretinal adhesion around retinal breaks is expected to form more quickly; thus, prolonged tamponade may be unnecessary [2, 9]. However, there remains controversy concerning the advantages of air tamponade with PPV for treatment of RRD with IRBs [8–11]. Thus, we investigated the anatomical and functional results of air tamponade for treatment of RRD with IRBs.

In the present study, the SSAS rate was 91.36% and the final success rate was 96.30%, respectively. These results are consistent with the findings in previous studies, where SSAS rates ranged from 76.9 to 93.3% [9, 16] and final success rates ranged from 95 to 100% [14, 17]. Furthermore, our patients exhibited improved visual outcomes at 1 week after surgery. We observed significant differences between preoperative BCVA and BCVA at both 1 month postoperatively and the last follow-up. In published literature regarding functional outcomes of RRD, a cut-off value for “good” visual function is often used. A threshold of 0.3 logMAR or 20/40 on the Snellen eye chart has been used in some studies; other studies demonstrated “good” visual acuity rates of 5 to 29% [18, 19]. In our study, more than half of the patients (56.8%) had BCVA < 0.3 logMAR at the last follow-up, although a large proportion of patients (81.48%) had macula-off retinal detachment.

Among the strengths of this study, all operations were performed by a senior chief physician and subretinal fluid was drained through retinal breaks with a flute needle during complete air-fluid exchange. Furthermore, retinal breaks were carefully identified by scleral depression. In previous research concerning air tamponade treatment for RRD, 360° prophylactic laser photocoagulation at the vitreous base was performed to reduce the risk of retinal redetachment from missed breaks; the incidence of secondary epiretinal membrane was 6.8% [10, 20]. In this study, 360° laser photocoagulation was not routinely performed. Only one patient (1.2%) in our study developed an epiretinal membrane.

Effective chorioretinal adhesion is a key aspect that prevents vitreous fluid from entering the subretinal cavity and causing redetachment. Compared with laser retinopexy, cryotherapy reportedly requires a longer interval for the formation of chorioretinal adhesion [8, 9, 21]. In the present study, cryotherapy alone was performed in one eye with a 4 DD horseshoe tear near the ora serrata; this patient had redetachment with reopening of primary retinal break at 1 month after the primary surgery. Thus, air tamponade may be unsuitable for such patients.

In a previous study, we demonstrated that adjustable postoperative positioning was safe and effective for RRD repair at various break locations [22]. Other studies found that RRD repaired by PPV plus air/gas tamponade could achieve high success rates when patients assumed the prone position for the first 24 h postoperatively or even without specific postoperative position [2, 8, 23, 24]. Angunawela et al. [25] evaluated fluid dynamics and fluid shear stress on the retinal wall in a model eye after vitrectomy and gas tamponade; their results suggested that restrictive posturing may be unnecessary and sudden head movements should be avoided. Thus, we advised the patients to assume a prone position during the first 24 h after surgery; this was followed by “support-the-break positioning” according to the locations of the breaks, which reduced postoperative discomfort.

Elevated IOP is a common postoperative complication that reportedly affects 58.9% of eyes with intraocular tamponade [4]. Elevated IOP after vitrectomy may cause optic nerve damage, retinal ischemia, and subsequent visual loss. In the present study, nine patients (11%) had IOP > 21 mmHg at 1 week postoperatively; the IOP returned to normal at 1 month postoperatively with short-term use of antihypertensive topical medication. Among the nine patients, five had axial length > 26 mm (range, 26.03–29.55 mm; mean, 27.56 mm), which suggests susceptibility to elevated IOP in patients with long axial length who receive intraocular air tamponade. A few studies have mentioned axial length as a risk factor for poor outcomes after gas/air tamponade [4]. Longer axial length may be associated with a more robust steroid response after topical ophthalmic corticosteroid treatment [26]. Therefore, topical ophthalmic corticosteroid was switched from dexamethasone to 1% fluorometholone 1 week after surgery in order to reduce IOP.

In multivariate analysis, Williamson et al. [27] found that breaks in the 5 to 7 o’clock hours had negative effects in patients with RRD. They also reported that the increased number of RRD-affected quadrants increased the risk of treatment failure; a greater number of breaks were closely associated with an increased number of detached quadrants. Goto et al. [15] found that IRBs represented the sole independent risk factor for redetachment, although their study excluded RRD with medium or large breaks and RRD with multiple breaks (both superior and inferior). In another study, air or sulfur hexafluoride was indicated for cases with single breaks that involved complete drainage of subretinal fluid; perfluoropropane was indicated for cases with large breaks, incomplete drainage of subretinal fluid, or multiple breaks [2]. In a pilot study of PPV plus air tamponade for RRDs with IRBs, the primary retinal reattachment rate and final reattachment rate were both very high (90 and 100%, respectively). However, that study excluded RRDs with > 5 breaks on preoperative examination [24], implying that multiple breaks in RRD was particularly challenging to manage by PPV alone. In our study, most eyes (61/81, 75.31%) had ≥ 2 breaks. In summary, 144 holes/breaks were found in the 5 to 7 o’clock region. Notably, 17 patients (20.9%) had > 5 retinal breaks; more than half of the breaks were medium or large. However, the number of retinal breaks was not a candidate risk factor for redetachment in our study (Table 4). Other studies also found that the number of detached retinal quadrants was an independent predictor of redetachment for RRD with IRBs; each additional clock hour involved in RRD was associated with a 12% increase in the risk of surgical failure [9, 28]. In the present study, the mean number of affected quadrants was 1.65 (range, 1–4 quadrants); this value was lower than in a previous study (3.83 quadrants) [10] and showed no association with surgical failure.

Because of anatomical characteristics, RRD surgery is challenging in eyes with high myopia; the SSAS rate in such eyes is reportedly lower than the SSAS rate in nonmyopic eyes. Janco et al. [29] found that the anatomical success of primary PPV for RRD did not differ between eyes with and without high myopia in patients with PVR grade A or B; however, only four (1.4%) patients underwent PPV with air tamponade in their study. In our study, 30 (37.03%) patients had axial length > 26 mm (range, 22.46–32.78 mm; mean, 25.52 mm); four of seven cases of retinal redetachment had high myopia with axial length ranging from 26.03 to 30.21 mm. In univariate analysis, axial length was identified as a candidate risk factor for redetachment (Table 4). Thus, PPV with air tamponade may be unsuitable for such patients.

It is difficult to find consistent evidence regarding risk factors for failed repair of primary RRD because of differences in study designs, inclusion criteria and surgical techniques. Patient age was identified as an independent risk factor for redetachment in our study population. The mean age of seven patients with retinal redetachments was 38.29 years, which is younger than that of patients without retinal reattachment. Park et al. found that people in the < 50 year age group accounted for the major part of increase incidence of RRD, which is related to the increasing incidence of myopia in the young generation in Asia [30]. In our study, both age and axial length were candidate risk factors for redetachment. It may also be associated with an increased incidence of myopia in younger patients.

This study had several important findings. First, our satisfactory anatomical and functional results indirectly provide clinical evidence to support the use of air tamponade in the treatment of RRD with IRBs. Second, our omission of 360° laser photocoagulation at the vitreous base may have contributed to the low incidence of epiretinal membrane (1.2%). Third, we found that both patient age and axial length were risk factors for redetachment in patients with IRBs; patient age was an independent risk factor for redetachment in our patients. However, randomized controlled clinical studies are needed to confirm our findings. Importantly, this study had several limitations, including its retrospective nature, small sample size, non-comparative design and non-randomized structure. We did not include a control group with long-acting gas tamponade because long-acting gases could not be used in China during the study period.

## Conclusion

Our findings show that air can be used as a tamponade in primary RRD with IRBs; such treatment provides satisfactory anatomical and functional results. Retinal surgeons should consider the utility of air tamponade for RRD with IRBs because this approach reduces the burden on patients, compared with long-acting gases or SO tamponade.

## Data Availability

All data generated or analyzed during this study are included in this published article.

## Abbreviations

RRD: Rhegmatogenous retinal detachment
IRB: Inferior retinal break
PPV: Pars plana vitrectomy
PVR: Proliferative vitreoretinopathy
SO: Silicone oil
SB: Scleral buckling
IOP: Intraocular pressure
BCVA: Best-corrected visual acuity
DD: Disc diameter
SSAS: Single surgery anatomical success
